# Data on prevalence and risk factors associated with *Toxocara* spp infection, atopy and asthma development in Northeast Brazilian school children

**DOI:** 10.1016/j.dib.2016.08.062

**Published:** 2016-09-17

**Authors:** Márcia B. Silva, Ana L.M. Amor, Leonardo N. Santos, Alana A. Galvão, Aida Y. Oviedo V, Eduardo S. Silva, Cynara Gomes Barbosa, Philip J. Cooper, Camila A. Figueiredo, Rita de Cassia Ribeiro, Neuza Maria Alcântara-Neves

**Affiliations:** a Instituto de Ciências da Saúde, Universidade Federal da Bahia, Salvador, Bahia, Brazil; b Centro de Ciências da Saúde. Universidade Federal do Recôncavo da Bahia, Santo Antônio de Jesus, Bahia, Brazil; c Faculdade de Farmácia, Universidade Federal da Bahia, Salvador, Bahia, Brazil; d Facultad de Ciencias Medicas, de la Salud y la Vida, Universidad Internacional del Ecuador, Quito, Ecuador; e St George’s University of London, Division of Clinical Sciences, Cranmer Terrace, London SW17 ORE, UK; f Escola de Nutrição, Universidade Federal da Bahia, Salvador, Brazil

**Keywords:** Risk factors, Toxocara spp, Atopy, Wheezing/Asthma

## Abstract

In the present article, we provide shortly, data on risk factors for acquiring *Toxocara* spp. infection and investigate possible associations between this infection with atopy and asthma in school children of a small town and its semi-rural areas of Northeast Brazil. The data set are composed by demographic, social and home environment variables. The detection of anti-*Toxocara* spp. IgG and specific IgE to aeroallergens was determined by ELISA and ImmunocAP/Phadiatrope systems, respectively. The data presented in this article are related to the article entitled “Risk factors for *Toxocara* spp. seroprevalence and its association with atopy and asthma phenotypes in school-age children in a small town and semi-rural areas of Northeast Brazil” (M.B. Silva, A.L. Amor, L.N. Santos, A.A. Galvão, A.V. Oviedo Vera, E.S. Silva et al., 2016) [1].

**Specifications Table**

**Table:** 

Subject area	Epidemiology, alergology
More specific subject area	Immunoparasitology
Type of data	Figure, Table
How data was acquired	ELISA, ImmunoCAP and Phadiatrope
Data format	Analyzed
Experimental factors	Stool and blood samples, and sera for measurement of allergen-specific IgE and anti-*Toxocara* spp. IgG
Experimental features	Immunoassay
Data source location	Federal University of Bahia, Salvador, Bahia, Brazil
Data accessibility	Data is available with this article

**Value of the data**

• These data set will be of value for the scientific community who work in the area of infectious diseases since it involves the risk factors related to *Toxocara* spp. infection.
• The data will also be of value for studies in the area of allergy and its interface with helminthic diseases, since they report an association of *Toxocara* spp. infection with aeroallergen specific IgE.
• These data reinforce the hypothesis that this association may be related to the cross-reactivity between parasite-specific and aeroallergen-specific IgE.

## 1 Data

**Fig. 1:**
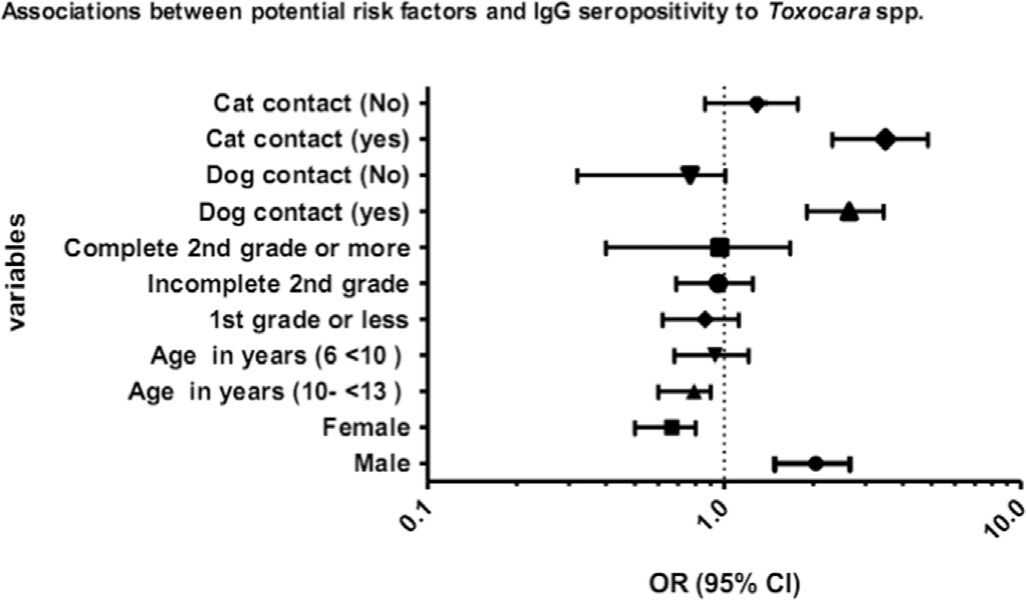
ORs and 95% confident intervals calculated by logistic analysis model adjusted by age, sex, contact with dogs and cats, school location, maternal schooling, family income and helminth infection.

The data demonstrate obtained in this work is summarized in Fig. 1 and Table 1. Fig. 1 shows that being male and having contact with dogs and cats were risk factors for *Toxocara* spp. infection among other variables studied. Table 1 shows the analysis of *Toxocara* spp. infection as risk factors for atopy and asthma. We found that *Toxocara* spp. seropositive school age children were more prone to have positive serum aeroallergen-specific IgE.

## 2 Experimental design, materials and methods

**Fig. 2:**
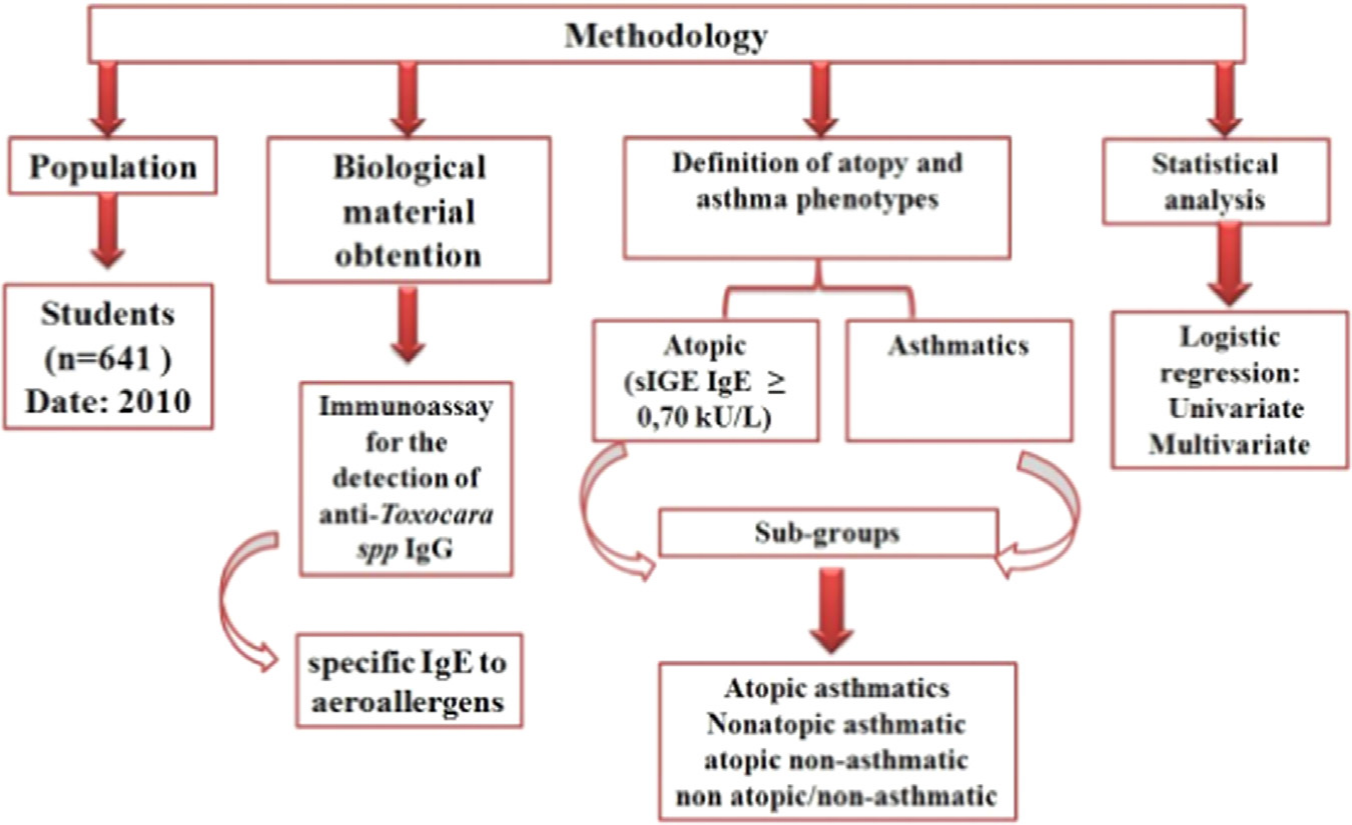
Schematic representation of the steps involved during the study to achieve the results.

The data presented in this paper investigated the risk factors to acquire *Toxocara* spp. infection and its association with atopy and asthma [1]. It reports the data obtained in a study conducted in a small city of Northeast Brazil with 791 school-age children. Students underwent an epidemiological survey answered by their parents on sanitation, social class and risk factors for toxocariasis. Moreover, they answered an ISAAC Portuguese-adapted questionnaire for asthma diagnosis [2]. Blood collection was performed to evaluate the levels of allergen-specific IgE and IgG anti-*Toxocara* spp. reactivity. Univariable and multivariable analyses were used to analyze*Toxocara* spp. infection risk factors and the association of this infection with atopy and asthma phenotypes. The variables investigated were: gender, age, maternal schooling, income, school location, contact with dog and cat (Fig. 2).

## Conflict of interest

The authors declare that they have no competing interests.

## Figures and Tables

**Table 1:** Association between *Toxocara* spp. seropositivity with atopy and wheezing/asthma in 791 elementary school students, 6–13 years old.

**Outcomes**	***n* (%)/*N***	**Adjusted OR (95% CI)**^a^
Phadiatop* IgE≥0.70 KU/L)	251(49.9)/503	**1.95 (1.40–2.72)**
***B. tropicalis*****specific IgE≥0.70 KU/L**	286 (56.8)/503	**1.85 (1.31–2.62)**
**Any allergen IgE≥0.70 KU/L**	342 (67.9)/503	**2.00 (1.49–2.68)**
**Atopic wheezing/asthma**	398(79.2)/503	1.04 (0.54–2.08)
**Non-atopic wheeezing/asthma**	57(11.3)/503	1.08 (0.40–2.70)

a IgE specific to *Blomia tropicalis* (D201) and to Phadiatop aerollergens (pollen extracts, fungi extracts, dog and cat epithelia and *Dermatophagoides* spp.) measured by immunoCAP.
