# Supplementary figures and images for: Data on prevalence and risk factors associated with Toxocara spp infection, atopy and asthma development in Northeast Brazilian school children
Source: Data Brief. 2016 Sep 17;9:425–8. doi: 10.1016/j.dib.2016.08.062 (PMC5037257; doi:10.1016/j.dib.2016.08.062)

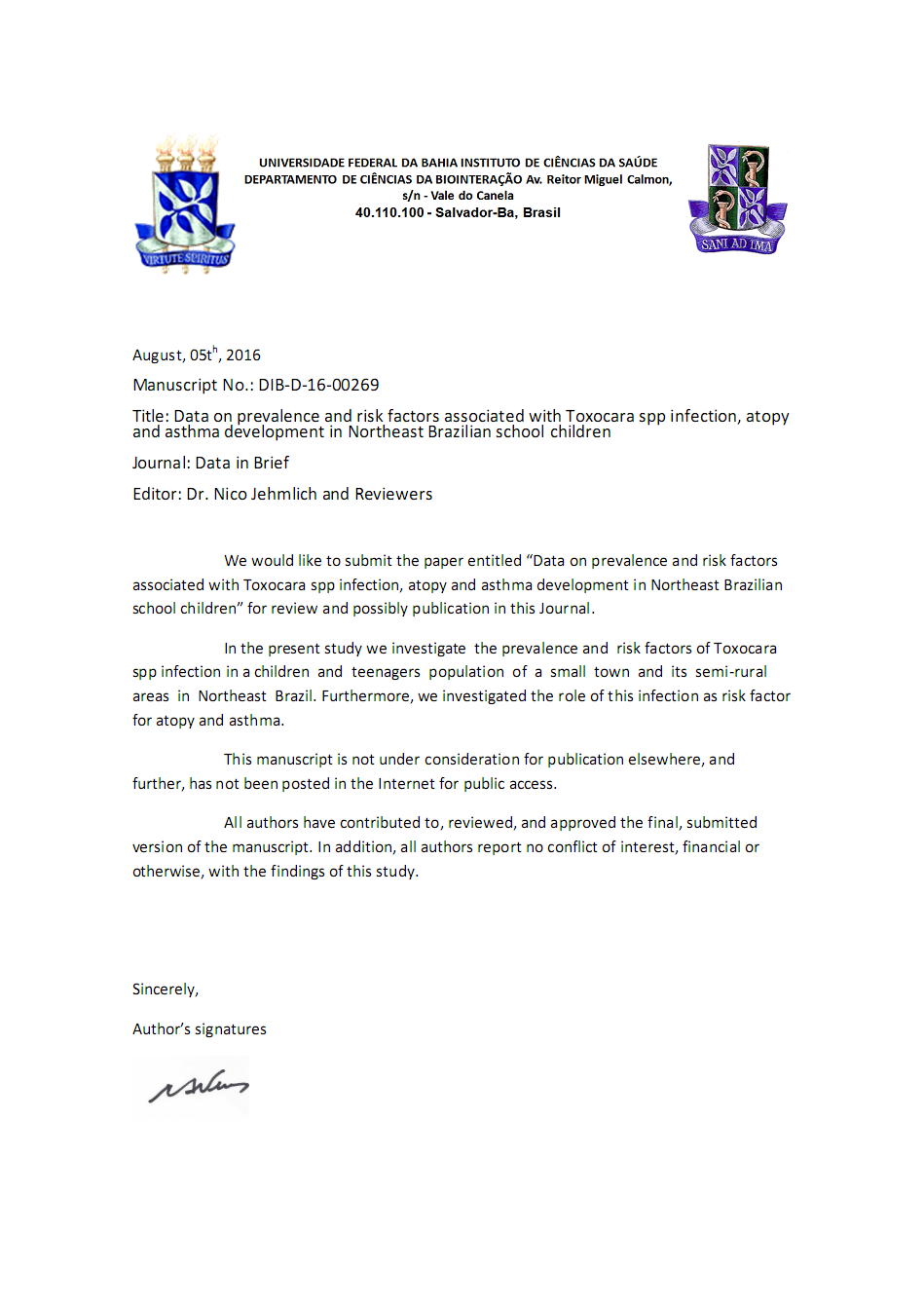


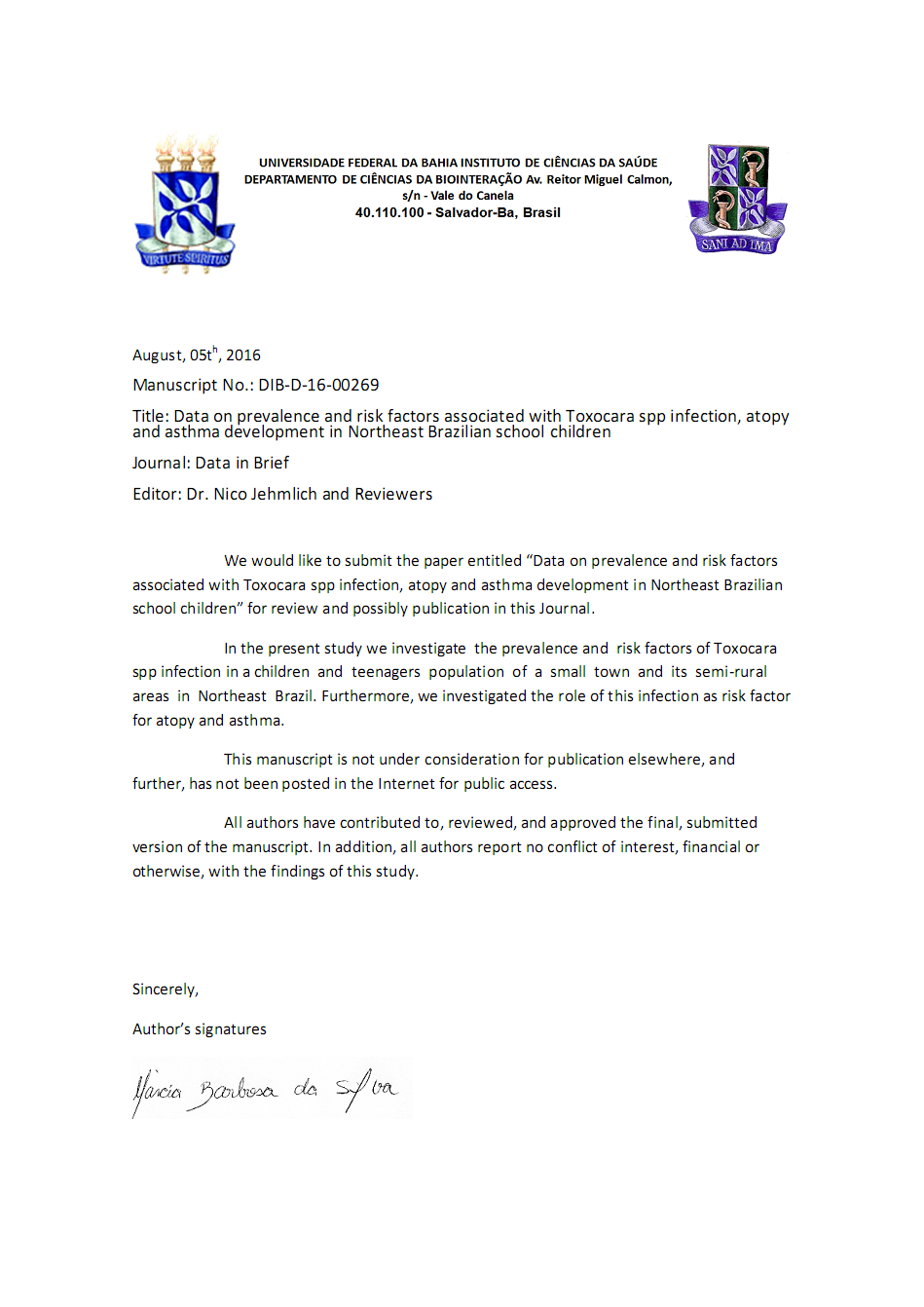


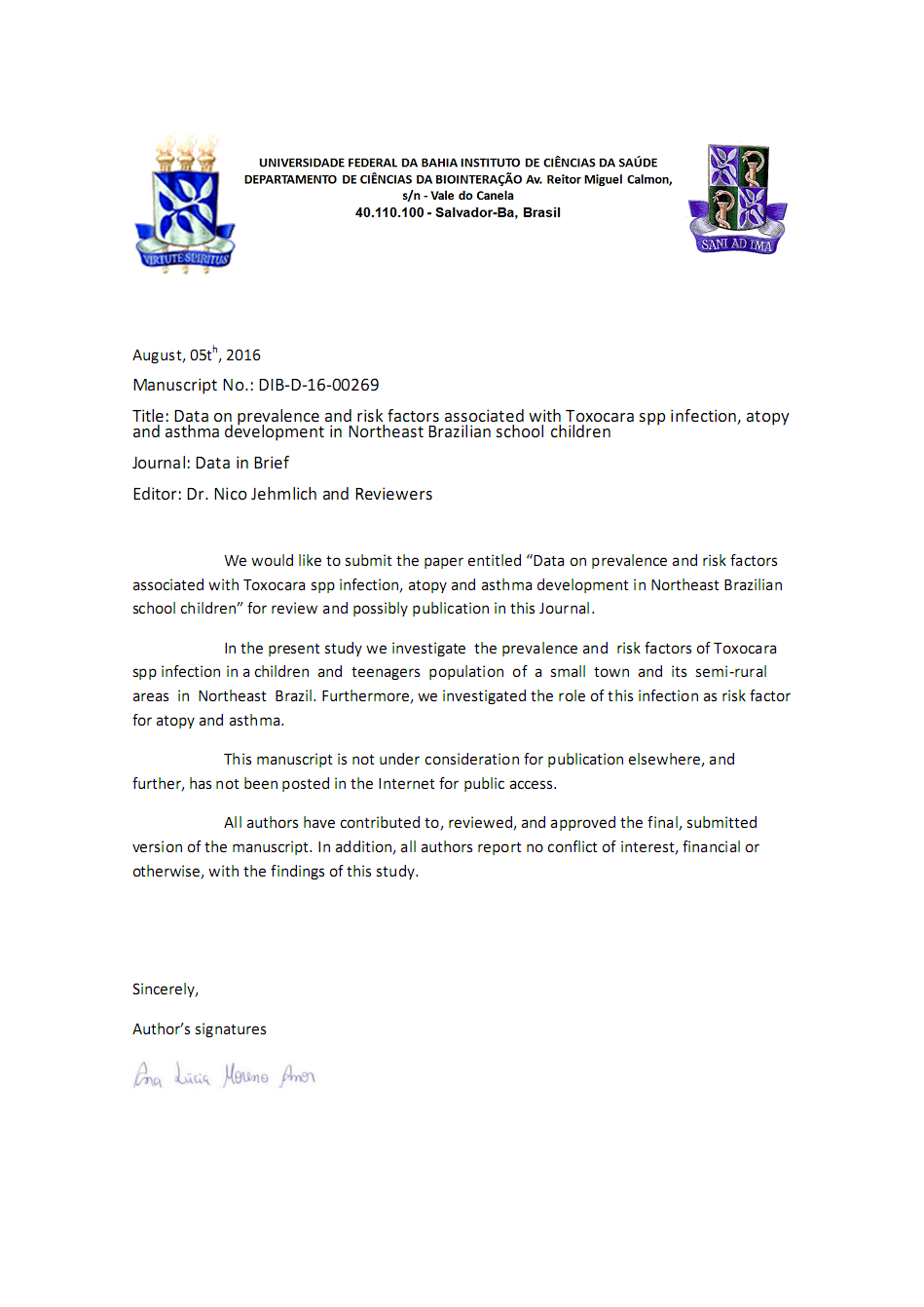


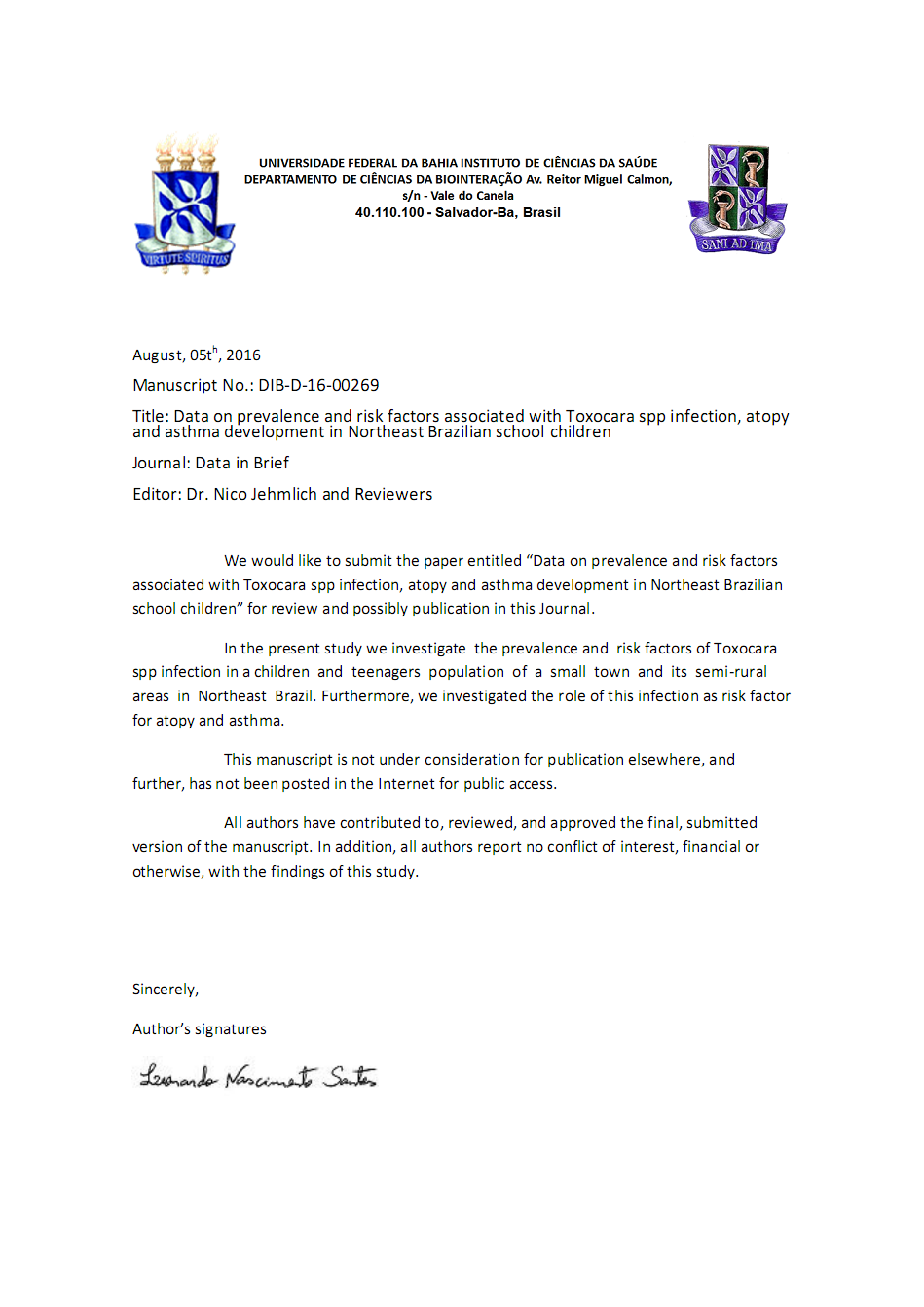


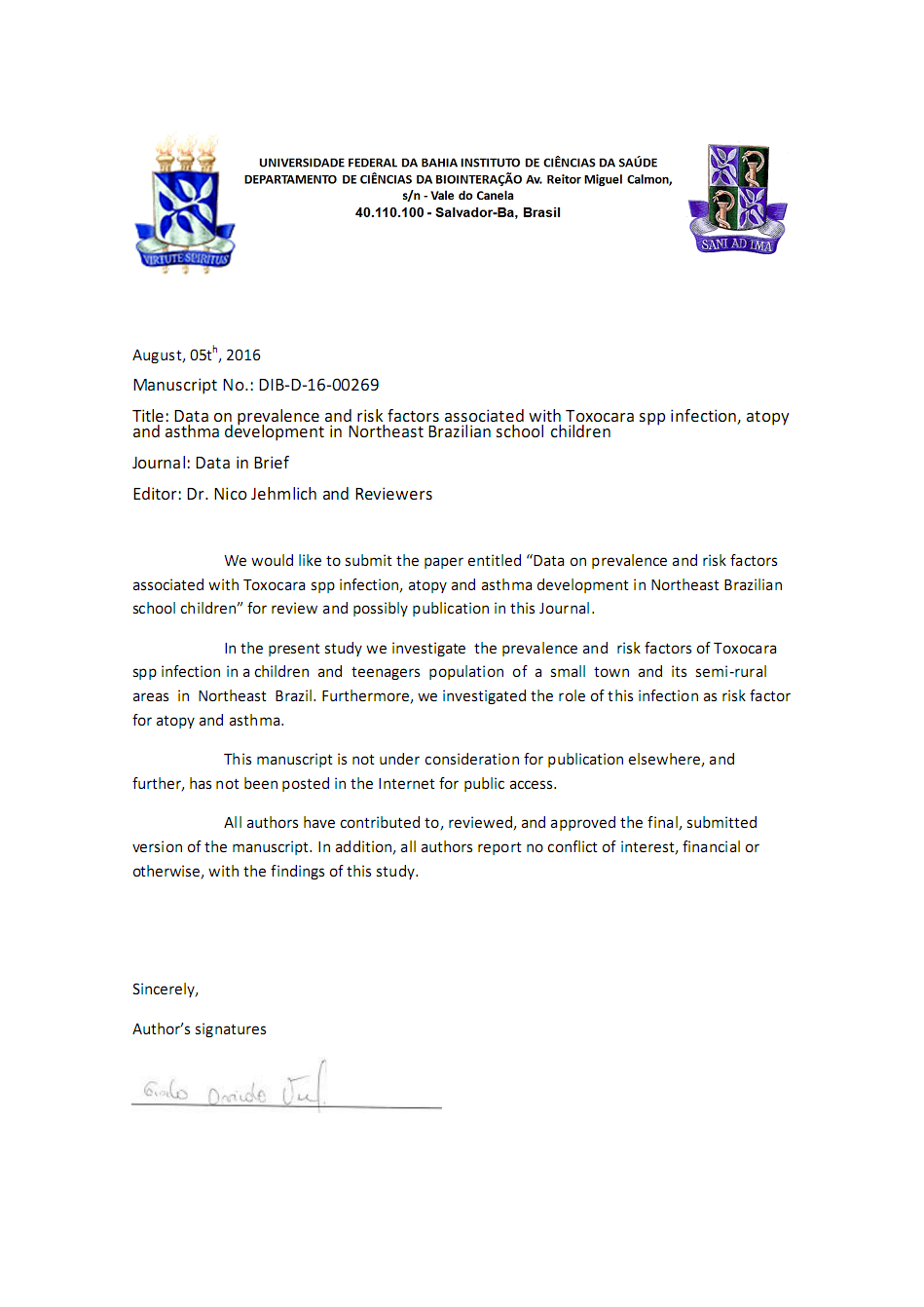


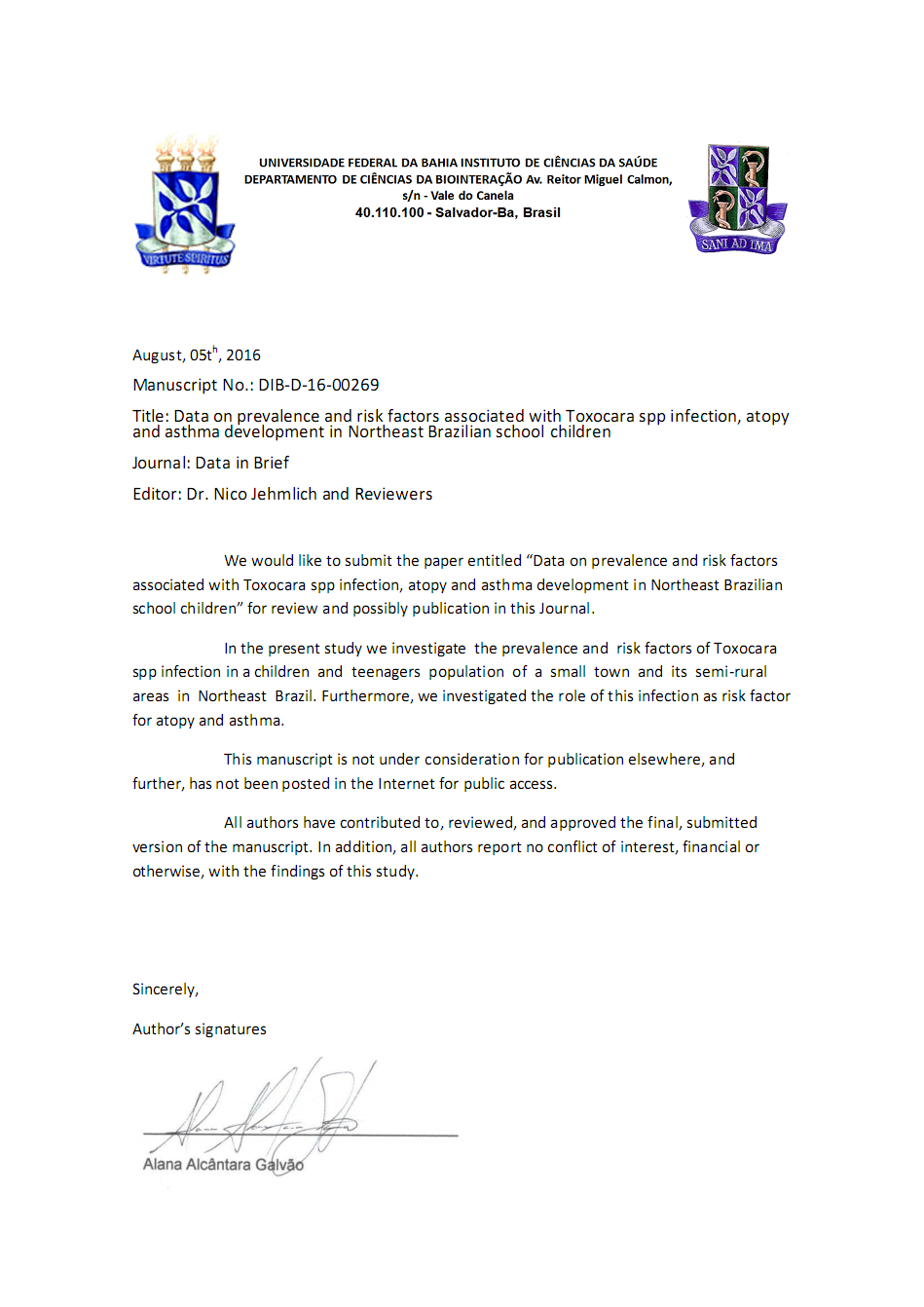


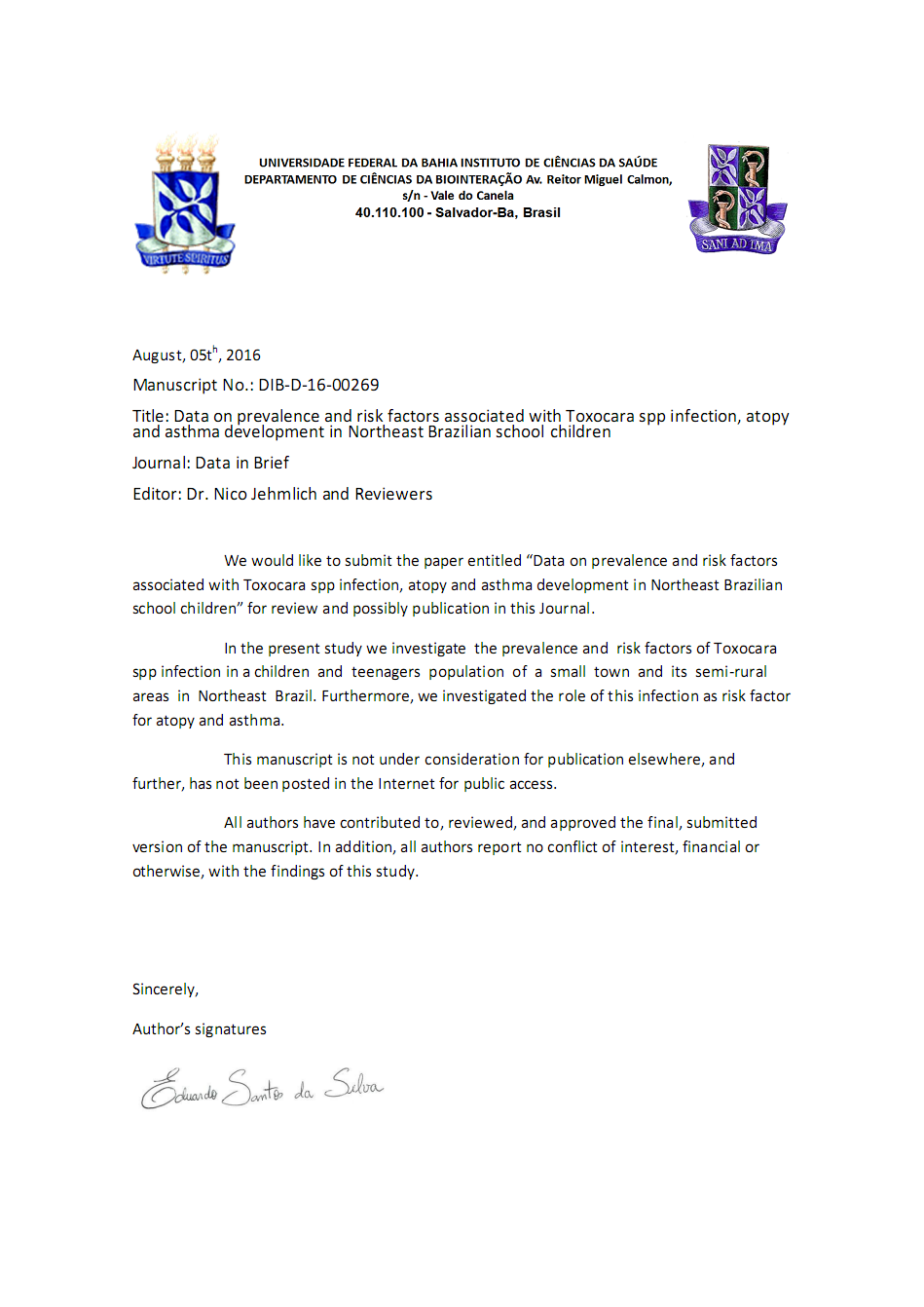


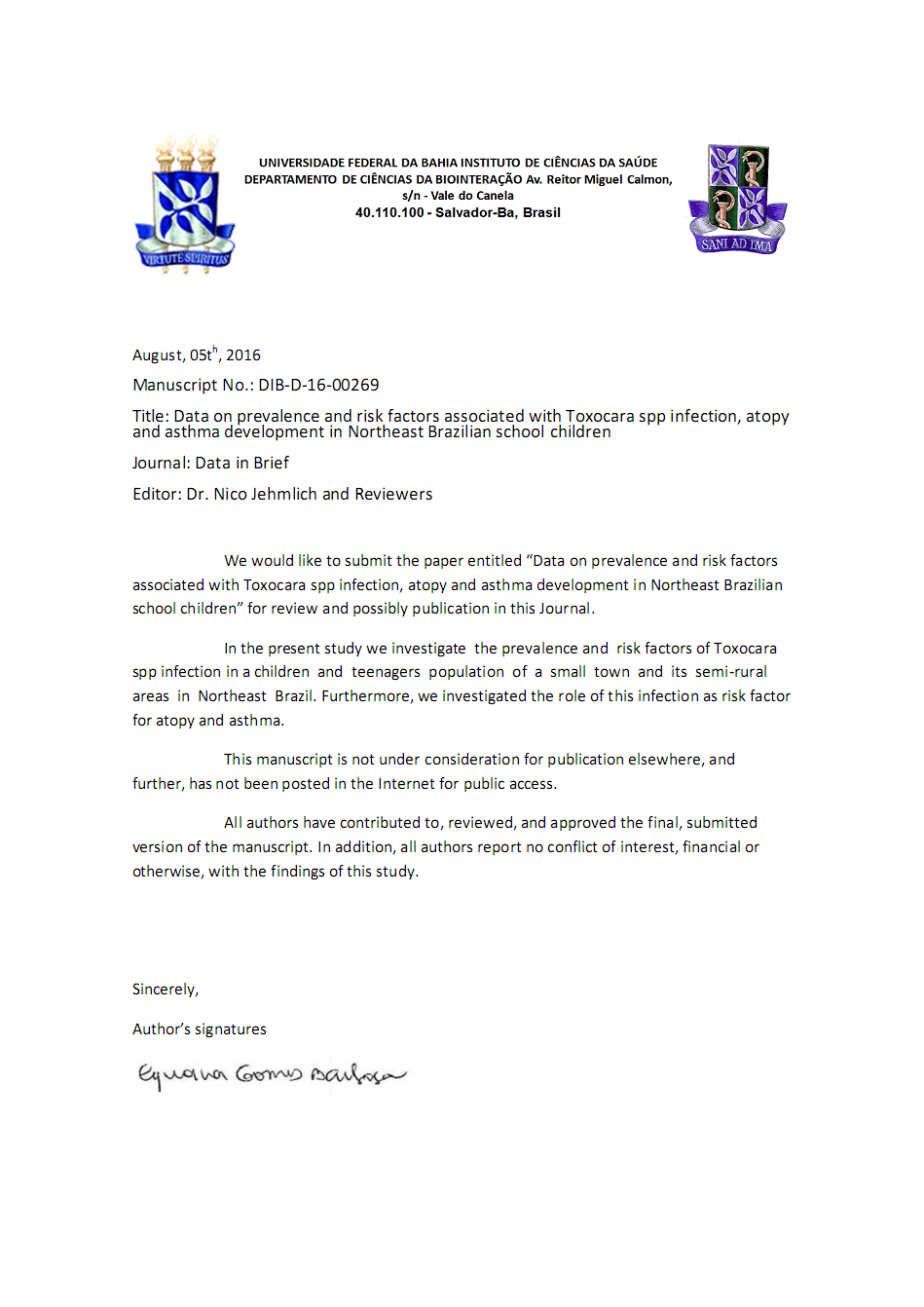


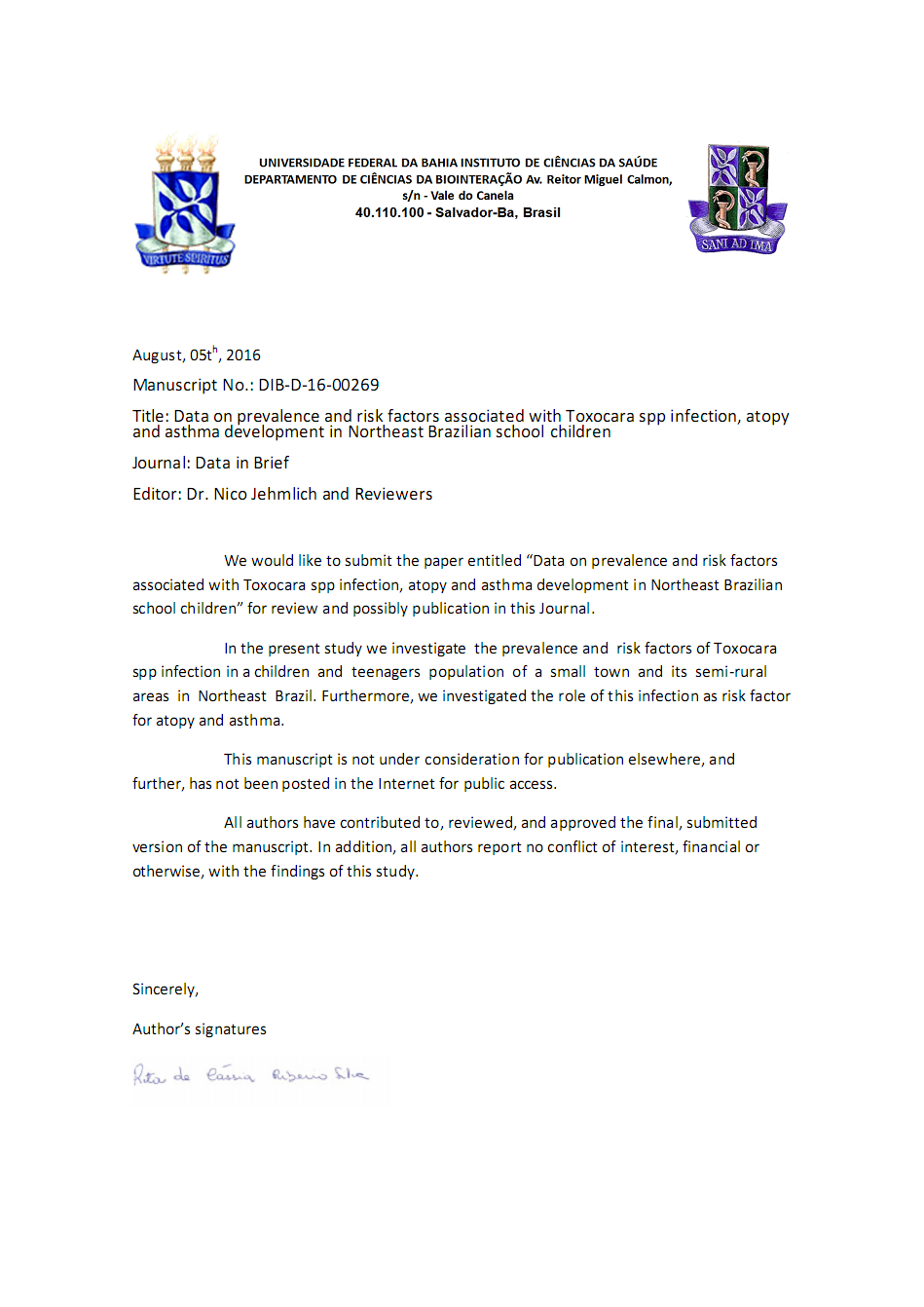


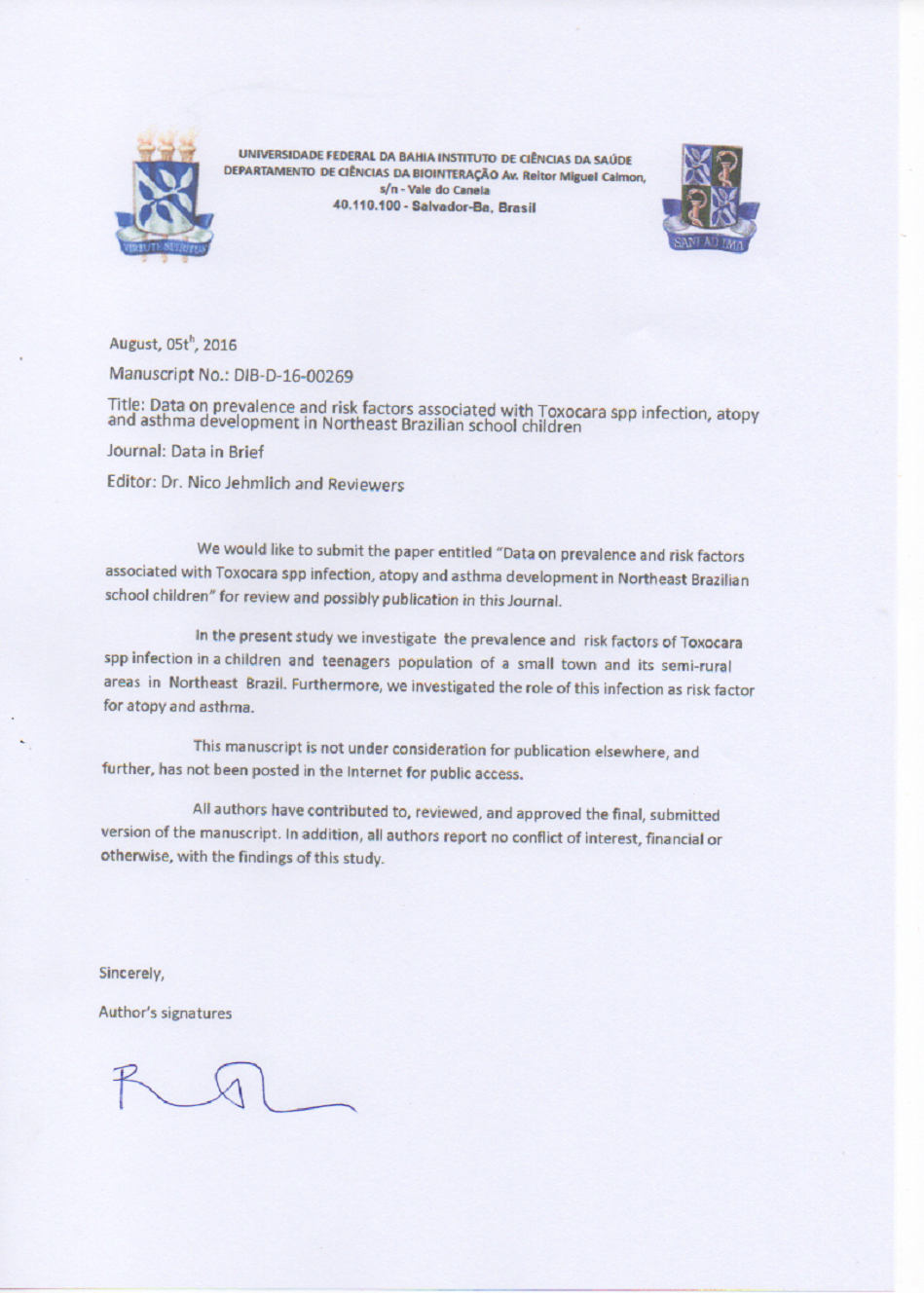


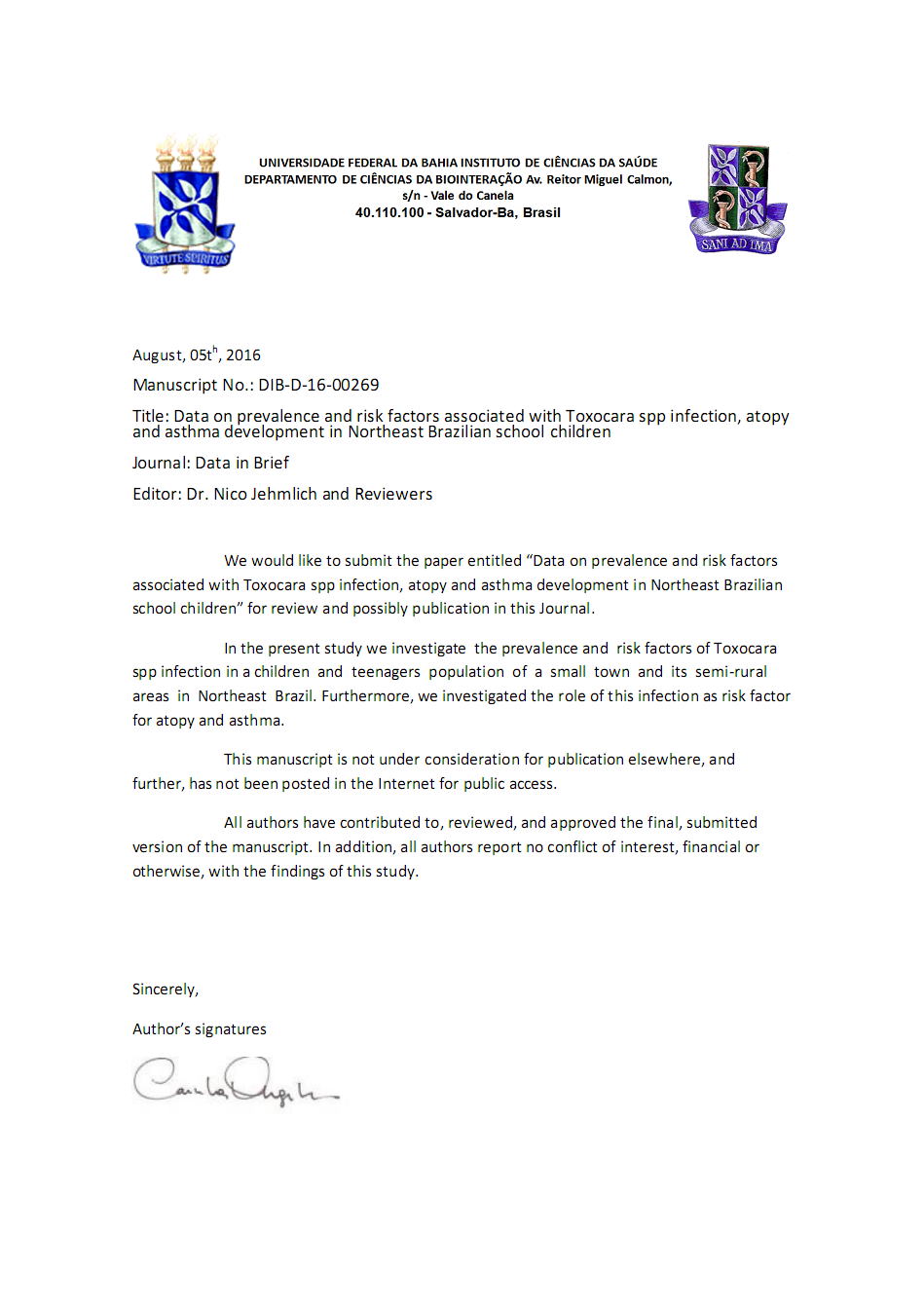

Supplement: Supplementary file 1 — Supplementary material [file mmc1.doc]
